# Expression and Secretion of Plasma Membrane Ca^2+^-ATPase 4a (PMCA4a) during Murine Estrus: Association with Oviductal Exosomes and Uptake in Sperm

**DOI:** 10.1371/journal.pone.0080181

**Published:** 2013-11-14

**Authors:** Amal A. Al-Dossary, Emanuel E. Strehler, Patricia A. Martin-DeLeon

**Affiliations:** 1 Department of Biological Sciences, University of Delaware, Newark, Delaware, United States of America; 2 Department of Biochemistry, Mayo Clinic, Rochester, Minnesota, United States of America; Clermont-Ferrand Univ., France

## Abstract

PMCA4, a membrane protein, is the major Ca^2+^ efflux pump in murine sperm where its deletion leads to a severe loss of hyperactivated motility and to male infertility. We have previously shown that the PMCA4b splice variant interacts with CASK (Ca^2+/^CaM-dependent serine kinase) in regulating sperm Ca^2+^. More recently we detected that PMCA4a isoform, in addition to its presence in testis, is secreted in the epididymal luminal fluid and transferred to sperm. Here we show that *Pmca4* mRNA is expressed in both the 4a and 4b variants in the vagina, uterus, and oviduct. Immunofluorescence reveals that PMCA4a is similarly expressed and is elevated during estrus, appearing in the glandular and luminal epithelia. Western analysis detected PMCA4a in all tissues and in the luminal fluids (LF) of the vagina (VLF), uterus (ULF), and the oviduct (OLF) collected during estrus. It was ~9- and 4-fold higher in OLF than in VLF and ULF, and only marginally present in LF collected at metestrus/diestrus. Fractionation of the LF collected at estrus, via ultracentrifugation, revealed that 100% of the PMCA4a resides in the vesicular fraction of the ULF and OLF. Transmission electron microscopy (TEM) revealed that OLF vesicles have an exosomal orientation (with the cytoplasmic-side inward), a size range of 25-100 nm, with the characteristic CD9 biomarker. Thus, we dubbed these vesicles “oviductosomes”, to which PMCA4a was immunolocalized. Incubation of caudal sperm in the combined LF or exosomes resulted in up to a ~3-fold increase of sperm PMCA4a, as detected by flow cytometry, indicating *in vitro* uptake. Our results are consistent with the increased requirement of Ca^2+^ efflux in the oviduct. They show for the first time the presence of oviductal exosomes and highlight their role, along with uterosomes and vaginal exosomes, in post-testicular sperm acquisition of PMCA4a which is essential for hyperactivated motility and fertility.

## Introduction

Capacitation which occurs in the female reproductive tract is the final maturational process that mammalian sperm undergo before they are competent to effect fertilization. At the fertilization site in the ampullary-isthmic junction (AIJ) [1], high intracellular Ca^2+^ concentration levels are essential for both hyperactivated sperm motility and the acrosome reaction [2]. PMCA4, a member of a family of Ca^2+^ efflux pumps, maintains low resting cytosolic [Ca^2+^] ([Ca^2+^]_c_) and a Ca^2+^ gradient across the sperm membrane and plays the major role in the maintenance of Ca^2+^ homeostasis in murine sperm [3]. The importance of PMCA4’s role in sperm is underscored by studies showing that global *Pmca4* knockout leads to a critical loss of progressive and hyperactivated motility, and subsequently infertility in mice [4,5]. 

We have previously shown that the PMCA4b splice variant interacts with CASK (Ca^2+^/CaM-dependent Serine Kinase) in regulating murine sperm Ca^2+^[6]. Recently we showed that PMCA4 splice variants 4a and 4b are secreted in the mouse epididymal luminal fluid and that 4a is transferred to the sperm membrane during epididymal maturation, with caudal sperm having a 5-fold increase over that in caput sperm [7]. Since caput sperm have 2-6 times higher [Ca^2+^]_c_ than caudal sperm and are incapable of progressive motility [8], the uptake of PMCA4a during epididymal transit may play a direct and vital role in sperm acquisition of progressive motility and in their viability. Similarly, it is likely that sperm may need to acquire additional PMCA4a in order to: a) preserve sperm fertility in the storage reservoir of the oviduct by avoiding premature capacitation, b) maintain viability after the demand for high intracellular [Ca^2+^] for hyperactivated motility in the AIJ [9], and for the acrosome reaction [2]. 

During transit in both the male and female tracts and during ejaculation, the sperm plasma membrane undergoes extensive modifications in which it acquires a variety of proteins [10]. Our laboratory has shown that SPAM1, a glycosyl phosphatidylinositol (GPI)-linked sperm membrane protein, is expressed in the oviduct and uterus where it is secreted and acquired by sperm [11,12]. We hypothesize that PMCA4a is also expressed and secreted in the female reproductive tract where it can be acquired by sperm during capacitation. Of the two splice variants of PMCA4, 4a shows a much higher basal activity and is more effective than 4b in returning Ca^2+^ to resting levels [13]. Thus it would readily meet the demands of maintaining homeostasis in conditions of high [Ca^2+^]_c_ during capacitation. Our results show that PMCA4a is expressed and secreted in the LF in the oviduct, uterus and the vagina, with the secretion highest in the LF of the oviduct where sperm are stored during estrus. Importantly, we detected that it resides in membranous vesicles in the OLF as well as in uterosomes from the ULF. Mature caudal sperm were shown to acquire PMCA4a *in vitro* after incubation in the LF or from their isolated exosomes. Our findings indicate that oviductal PMCA4a may play an important and crucial role in the regulation of proper Ca^2+^ handling during sperm capacitation. 

## Materials and Methods

### Animals and Reagents

Sexually mature 4-12 week old female and 3-6 month old male mice (C57BL/6 and ICR strains; Harlan, Indianapolis, IN) were used throughout the investigation. In addition to these wild-type (WT) mice, *Pmca4* null mice [4] were used to provide epididymal tissue for the Western blotting analysis. These mice were a generous gift from Dr. Gary Shull in whose laboratory they were generated. Breeding and genotyping of these mice were described previously [4]. Studies were approved by the Institutional Animal Care and Use Committee at the University of Delaware and were in agreement with the Guide for the Care and Use of Laboratory Animals published by the National Research Council of the National Academies, 8th ed., Washington, D.C. (publication 85-23, revised 2011). 

### Antibodies

Rabbit polyclonal antibodies against peptides specific for bovine PMCA4a have been generated and previously validated [14,15]. Sequence analysis suggested a high probability of cross-reactivity with mouse PMCA4a, and studies in male mice revealed the specificity of the antibodies for this species [7]. Thus these antibodies were used in our Western blots, immunofluorescence, immunoelectron microscopy, and flow cytometric studies, all of which further demonstrated their competence to specifically detect mouse PMCA4a. Anti-CD9 antibody (SC-18869) and anti-human HSC70 (heat shock cognate protein 70; SC7298) mouse monoclonal antibody were obtained from Santa Cruz Biotechnology, and β-actin antibody (#4970) from Cell Signaling (Boston, MA).

### Superovulated Females

Four to 6-week-old C57BL/6 females were induced into estrus by sequential administration of pregnant mare serum gonadotropin (7.5 i.u.) and human chorionic gonadotropin (7.5 i.u.) (Sigma-Aldrich, St. Louis, MO) spaced 48 h apart. Reproductive tissues (uterus, vagina, oviduct) were removed, after sacrificing females 13.5-14 h following the last hormonal injection. 

### Preparation of Murine Female Reproductive Tissues and Isolation of Caudal Sperm

Vagina, uterus and oviduct were dissected immediately after sacrifice and frozen at -80°C for subsequent expression analysis, or they were flushed with PBS to remove the LF and immediately frozen at -80°C. Sperm were harvested from the caudal epididymides of 4-6 sexually mature males per experiment, by mincing the caudae in PBS with Protease inhibitor as described earlier [16] and kept at 37°C for 10 min to allow sperm to swim out of the tissue. After gravity settling of the tissues the supernatant was collected and subjected to centrifugation at 500 x *g* for 10 min to pellet the sperm which were then washed with PBS.

### Total RNA Isolation and Reverse Transcriptase (RT)

PCR*-*Reproductive tissues (vagina, uterus, oviduct) were collected from two virgin females after superovulation, and total RNAs were extracted using RNeasy Mini Kit (Qiagen, Valencia, CA) according to the manufacturer’s directions and RNA samples were further treated with DNAase (Turbo DNA-Free, Ambion Inc., Austin, TX). Total RNA (1μg) was used to synthesize cDNA using the iScript^TM^ cDNA synthesis kit (BioRad, Hercules, CA). Samples without the addition of reverse transcriptase served as controls. To amplify cDNA samples, PCR reactions (25 µl final volume) were performed with 2 µl cDNA for 30 cycles (ABI Veriti Thermocycler), using *Pmca4*a and *4b* primers as follows: forward primer sequence was 5'- GGA CGA GAT TGA CCT TGC CG -3' and the reverse 5'- CAC CAT CCA ACA GGA GCA CAC T-3'. PCR amplification was performed for 35 cycles at denaturation for 30 sec at 96°C, annealing for 30 sec at 59°C, extension for 1 min at 72°C, and a final extension of 5 min at 72°C. *Gapdh* was used as an internal control, Using commercial mouse *Gapdh* primers (Qiagen, Valencia, CA), PCR amplification was performed for 34 cycles at denaturation for 10 sec at 95°C, annealing for 30 sec at 55°C, extension for 90 sec at 72°C, and a final extension of 10 min at 72°C. The total RNA extracted from testis was used as positive control. RT-PCR products (10 µl) were run on 2% Nusieve agarose gel in TAE (40 mM Tris-acetate, 2 mM EDTA, pH 8.5) containing ethidium bromide (10 µg/ml), along with a 100 bp ladder (Invitrogen). 

### Protein Localization by Immunofluorescence (IF)

Mouse vaginal, uterine, and oviductal tissues were collected from superovulated females and immediately embedded in optimum cutting temperature media (OCT) (Tissue Tek, Torrance, California) and frozen at -80°C, or were frozen immediately after flushing to remove he luminal fluids. Cryostat sections (20μm) were made and kept in -80°C until processing. Slides were fixed in 1:1 acetone:methanol pre-chilled for 20 min at -20°C and then allowed to air-dry for 10 min before being placed in protein blocker [1% bovine serum albumin (BSA) in PBS] for 1-2 h at RT. They were then incubated overnight at 4°C with the anti-PMCA4a primar*y* antibody diluted at 1:50 in blocking solution, or with PBS or rabbit IgG for the negative controls, followed by washing with PBS (2X, 20 min). Sections were then incubated for 1h at RT with Alexafluor 568-conjugated goat anti-rabbit IgG (Molecular Probes, Eugene Oregon, 1:200) containing 1:2000 Draq-5 as a nuclear stain (Biostatus Limited, Leicestershire, United Kingdom), followed by (2X, 20 min) washing with PBS. Finally, the samples were mounted with mounting media and cover-slipped. Slides were visualized using a Zeiss LSM 780 confocal microscope (Carl Zeiss, Inc, Gottingen, Germany) using a plan-Apochromatic 20x objective. 

### Analysis of PMCA4a Expression during Physiological Estrous Cycle

Eight to 12 week-old virgin females were used in this investigation. They were categorized by the stages of estrous cycle based on the proportion of different cell types observed in the vaginal secretion [17]. Uterine tissues were collected from females at diestrus, proestrus, estrus, and metestrus. Tissues were then processed for IF as described above. 

### Collection of Luminal Fluids in Naturally Cycling Females and from Males

Caudal luminal fluids, used as controls, were collected from the epididymal caudae pooled from 2-3 WT ICR mice, using a method that was previously described [7,18]. Reproductive luminal fluids were collected from 4 females in proestrus and estrus (combined), and from 4 in metestrus and diestrus (combined), as previously described [12,18]. Briefly, vaginas, uterine horns and oviducts were flushed with PBS plus protease inhibitors by inserting a 26-gauge needle attached to a 1 ml syringe into the lumen and fluids were collected. Centrifugation at 3,500 x *g* for 10 min was used to exclude tissue fragment and cells and to clarify the female fluids. 

### Collection of Vaginal, Uterine and Oviductal Luminal Fluids from Females after hormonal induction of Estrus

VLF, ULF and OLF were obtained from 6-8 superovulated female mice per experiment. The methodology of collecting the fluid is similar to described above [18].

### Fractionation of ULF and OLF

Clarified uterine and oviductal luminal fluids, were collected from 6-8 superovulated female mice as described above. To fractionate the female LF (FLF) we used a methodology similar to previously described [12]. Briefly, the clarified FLFs were subjected to ultracentrifugation at 120,000 x *g* for 2 h, at 4°C using a Beckman Optima 2-70 k ultracentrifuge and a Ti60 rotor. The resulting pellets were re-suspended in homogenization buffer and protease inhibitor, for Western blot analysis, or in PBS to a final concentration of 2 mg/ml proteins to be used for co-incubation with sperm or transmission electron microscopy (TEM). The supernatants were collected and the proteins precipitated with 10% TCA and recovered in sample buffer, before being subjected to SDS-PAGE and Western blotting. 

### SDS-PAGE and Western Blot Analysis

Preparation of protein extracts from vagina, uterus, and oviduct was performed as described previously [11], using caudal sperm as a positive control. Total protein concentration in the lysates was determined using the bicinchoninic acid protein assay Kit (Pierce), according to the manufacturer’s protocol. Samples for electrophoresis were diluted in 2x Laemmli sample buffer with DTT (final concentration 100 mM) and urea (125 mg/ml) and incubated for 10 min at 37°C. Twenty to 60 µg of proteins from tissues and fluids, respectively, were loaded per lane on 10% polyacrylamide gels and transferred onto a nitrocellulose membrane (Amersham Biosciences). Western blotting was performed with the WesternBreeze Chemiluminescent Immunodetection Kit (Invitrogen) according to the manufacturer’s instructions. Blots were blocked for 1h at RT and incubated in anti-PMCA4a primary antibody (1: 500) or in anti-CD9 (1:1000) primary antibody overnight at 4°C. Non-specific binding of antibody was removed using 5X washes of TBST (20 mM Tris, pH 8.0, containing 150 mM NaCl and 0.5% Tween 20) before incubation in the alkaline phosphatase (AP)- conjugated anti-rabbit IgG (Invitrogen, diluted 1:2000) or AP-conjugated anti-rat IgG (Sigma-Aldrich, diluted 1:32,000) for 1 h at 4°C. The membrane was again washed (6X, 15 min) using TBST before chemiluminescence was detected by using the ECL kit (Bio-Rad, Hercules, CA). The membrane was re-probed with HSC70 or β-actin antibody which was used as internal loading control and for normalization. 

### Negative Staining for TEM

Nickel TEM grids (Electron Microscopy Sciences), 400 mesh with a formvar/carbon film, were floated on a drop of the fractions of purified OLF pellet suspension. The grids were then washed with several drops of water and then stained with 1% uranyl acetate, a phospholipid stain, before being subjected to microscopic analysis. Membrane vesicles were imaged using the TEM (Zeiss LIBRA 120). 

### Immunogold Labeling of PMCA4a or CD9 in Exosomes

The methodology of immunogold labeling is similar to previously described [19]. Briefly, following ultracentrifugation, vesicle suspensions were mixed 1:1 with 4% paraformaldehyde in 0.15 M Sorensen’s phosphate buffer pH 7.4 then were applied to 400-mesh nickel grids. The grids were then washed on 3 drops of PBS then incubated with 0.05 M glycine in PBS for 15 min. After blocking with goat block (Aurion, Wageningen, Netherlands) for 30 min and washing, the grids were incubated with primary anti-PMCA4a antibody diluted 1:10 in 0.1 % BSA-c^TM^ containing 0.02 % Triton X-100 (to permeabilize the vesicle membranes) for 2 h or CD9 antibody diluted 1:10 in 0.1 % BSA-c^TM^. Grids were then washed 6X, 5 min each, with 0.1 % BSA-c^TM^ (Aurion) and then were exposed to goat anti-rabbit or goat anti-rat secondary antibodies conjugated to 6 nm gold particles (Aurion) diluted 1:20 in 0.1 % BSA-c^TM^ (Aurion) for 2 h. The grids were then washed 6X, 5 min each, with 0.1% BSA-c and then with PBS 3x, 5 min each. Grids were post-fixed with 2% glutaraldehyde in PBS for 5 min and then washed with PBS for 5 min, followed with filtered double de-ionized water 5X, 2 min each. The membrane vesicles on the grids underwent negative staining with 1% uranyl acetate. After drying, the grids were examined with a Zeiss LIBRA 120 electron microscope operated at 120 kV. Control samples were processed identically, except that rabbit IgG or rat IgG was substituted for the primary antibody. 

### Analysis of *In Vitro* Sperm Uptake of PMCA4a from Female Luminal Fluids (FLF)

*In Vitro* sperm uptake assay of PMCA4a was similar to that previously described from our lab [7,12,16,18]. Briefly, caudal sperm were incubated in exosomes or unfractionated FLF (combined VFL, OFL and ULF), recovered as described above in PBS supplemented with 200 μM zinc acetate and protease inhibitor, pH 7.0. This PBS-zinc acetate solution was used for incubating equal aliquots of control sperm samples. After co-incubation of sperm and FLF for periods of 2-3 h at 37°C, control and test samples were washed (3X, collected with centrifugation at 500 x *g* for 15 min) using PBS with 200 μM zinc acetate. They were then fixed with 1.5% paraformaldehyde for 1 hr at RT, washed (3X, 15 min) with PBS and then permeabilized with 0.1% Triton X-100 for 10 min at RT. After washing cells with blocking buffer (2% BSA in PBS) they were incubated in blocking buffer for 30 min at RT. Permeabilized cells were then incubated in anti-PMCA4a primary antibody (1:200 dilution in blocking solution, applied to the cells at 4°C, for 1h), followed by washing (3X, 15 min) with PBS. Alexafluor 488-conjugated donkey anti-rabbit IgG (Molecular Probes, Eugene Oregon) diluted 1:200 in blocking solution was used as a secondary antibody in which cells were incubated at RT for 30 min in the dark, followed by washing (3X, 20 min) with PBS. Stained sperm were analyzed by flow cytomety on a FACSCalibur or a FACSAria^TM^ II (BD Sciences, San Jose, CA), equipped with an argon laser at 488 nm excitation. Analysis was performed using standard protocols and quantification criteria to assess PMCA4a uptake by sperm.

### Statistical Analysis

Two-way ANOVA and Student’s *t*-tests were performed on the means *±* **±**SEM for 3 replicates, to compare the statistical significance between unpaired data or for multiple groups, respectively. *P* values were calculated and *P<0.05 was considered significant. 

## Results

### Analysis of *Pmca4* Transcripts in Murine Female Reproductive Tissues

**Figure 1:**
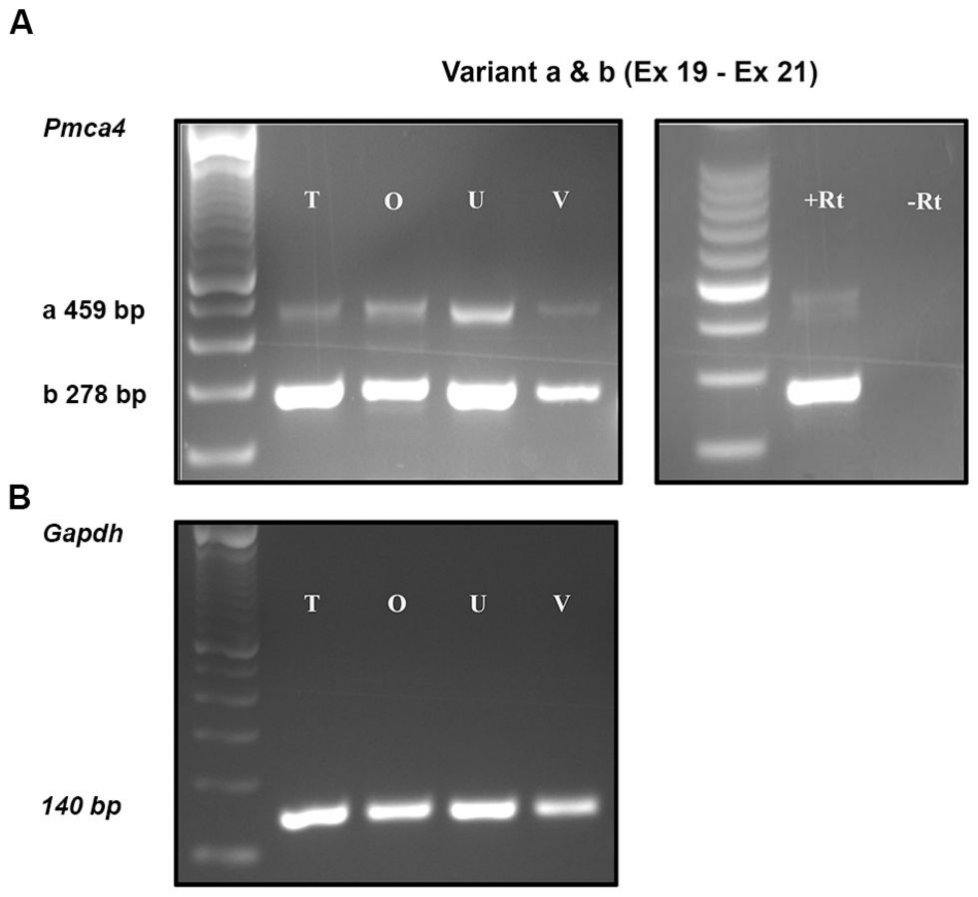
Semi-quantitative analysis of *Pmca4a, b* mRNA via RT-PCR in the murine female reproductive tract **A**) The 469 bp PCR product represents *Pmca4a*, and that of 278 bp corresponds to *Pmca*4b. Murine testis (T) cDNA served as positive control. Negative control was performed in the absence of reverse transcriptase (-RT). *Gapdh* mRNA was used as an internal control (**B**). Pronounced expression of *Pmca4b* was detected in the uterus, whereas PMCA4a showed more prominent expression in the oviduct than in the testis. A 100 bp ladder was run in the left lane of each panel. O, oviduct; U, uterus, and V, vagina.

To obtain information on the expression of transcripts from the C-terminal splice variants of *Pmca4* in the murine female reproductive tract, we performed RT-PCR using specific primers. Testis tissues were used as a positive control. As shown in Figure 1 A, mRNA for *Pmca4*a and *4b* variants was present throughout the female tract in tissues recovered following superovulation. The expected PCR products of 469 and 278 bp were observed in all three regions: the oviduct, uterus, and vagina. The mRNA distribution varied in the tissues. Of the two variants *Pmca4b* mRNA is more abundant in all three regions (Figure 1B). Sequence analysis of the 469 and 278 bp PCR products matched the cDNA sequence of PMCA4a and the alternatively spliced PMCA4b, respectively. A negative control for the PCR showed no product for the reaction in the absence of reverse transcriptase. 

### Localization and Detection of PMCA4a in the Female Reproductive Tract and in its Secretions

**Figure 2:**
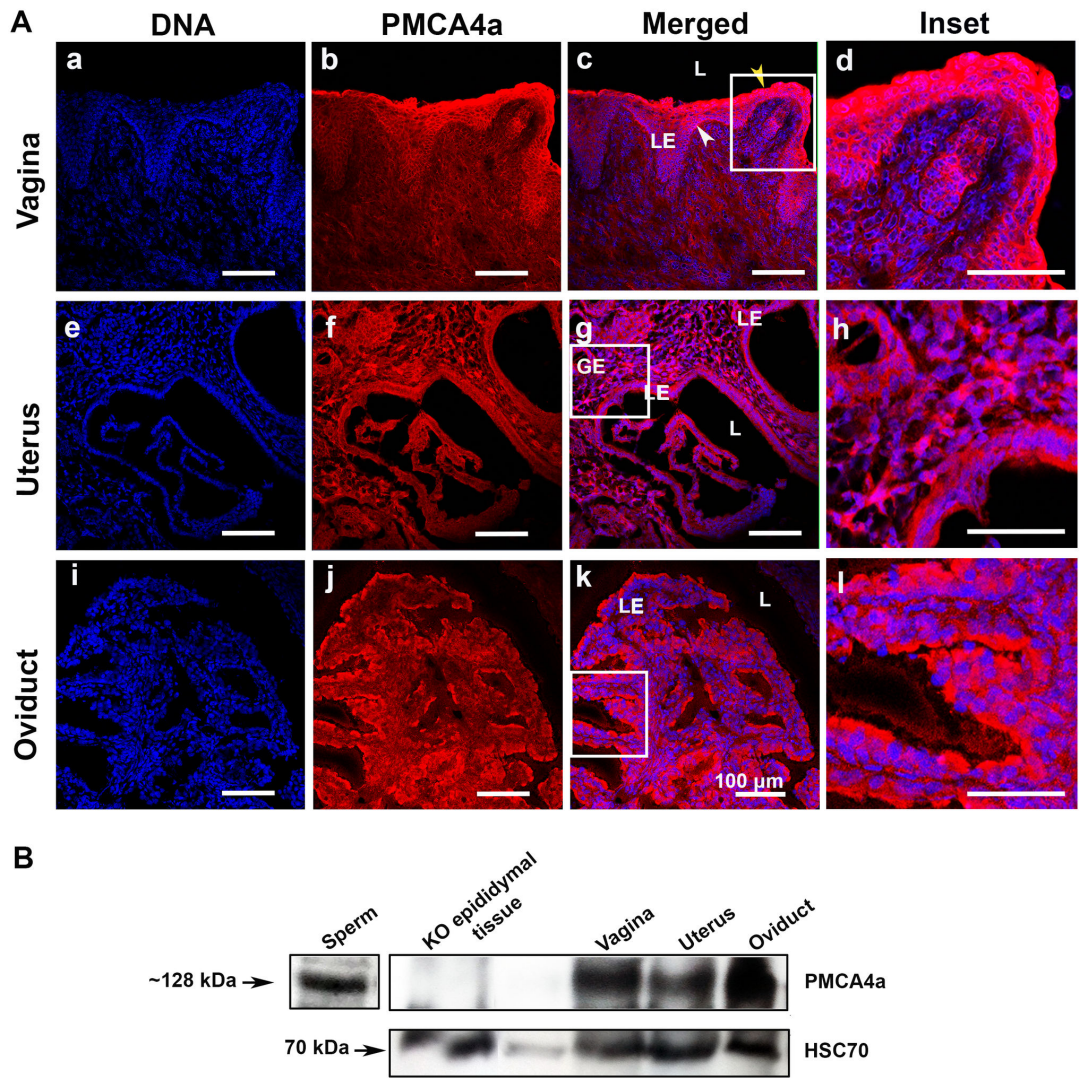
Expression of PMCA4a in mouse vaginal, uterine, and oviductal tissues following superovulation **A**) Indirect immunofluorescence was performed on frozen sections (**a**-**d**) of vaginal tissues using anti-PMCA4a antibody and an AlexaFluor-conjugated secondary antibody (red), and the nuclei were visualized by staining with Draq-5 (blue). Strong PMCA4a staining was detected at the epithelial layers at the luminal edge (yellow arrowhead) and was decreased at the basement membrane (white arrowhead). (**e**-**h**) In uterine tissue PMCA4a was abundantly expressed in both the luminal and glandular epithelia and also in the stroma. (**i**-**l**) In oviductal tissue strong PMCA4a staining is detected at the apical boundaries of the epithelial cells that line the oviductal lumen. (**d**, **h**, and **l**) Insets are seen from vagina, uterus and oviduct. Negative controls in PBS or IgG of the tissues showed no staining, similar to that in Figure 3, 4. The images were captured using confocal microscopy with a 20x objective lens (a plan-Apochromatic). L= lumen; LE = luminal epithelium; GE = glandular epithelium. Bar = 100 µm (same scale for all micrographs, and 200 µm insets). **B**) Western blot analysis performed with anti-PMCA4a antibody on tissues recovered after superovulation, using sperm as a positive control, revealed the ~128 kDa PMCA4a in all tissues and its absence in epididymal tissues of *Pmca4* null mice, used as a negative control (Top panel). In the lower panel, equal loading of protein is demonstrated by detection of HSC 70 in the tissue lysates. The amount of proteins loaded was 20 μg per lane.

To determine the expression and localization pattern of the PMCA4a protein in the female reproductive tract, sections of vaginal, uterine, and oviductal tissues from superovulated virgins were subjected to indirect IF analysis using the polyclonal PMCA4a antibody. The results in Figure 2A show that PMCA4a is mostly present in the luminal and glandular epithelial cells of the endometrium of the uterus. In the vagina a strong signal of PMCA4a was detected in the mucosa and within the epithelial layers at the apical membrane near the lumen (yellow arrowhead) and a weaker staining at the basement membrane (white arrowhead). In the oviduct the signal for PMCA4a is strongest in the luminal epithelia of the convoluted epithelial lining on the apical membrane. The presence of PMCA4a on the apical membrane of the luminal epithelial cells suggests that PMCA4a is secreted in the lumen. 

**Figure 3:**
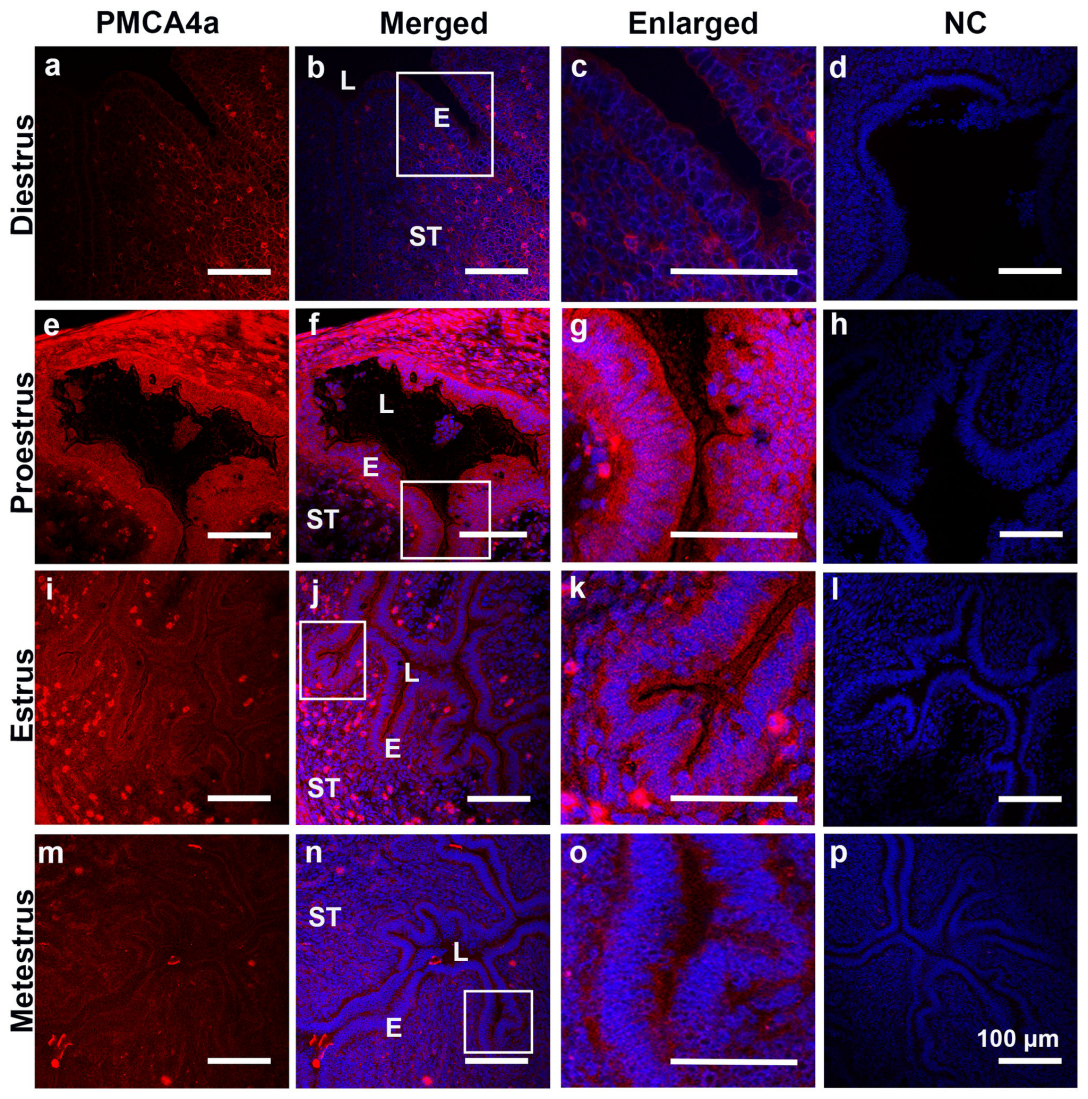
Indirect Immunofluorescence of PMCA4a in the murine endometrium of the uterus during the estrous cycle Using frozen sections PMCA4a immunoreactivity (red) was detected in the uterine luminal epithelium in pro-estrus (e - g) and estrus phases (i - k) but not during metestrus (m - o) and diestrus (a - c). The nuclei were visualized by staining with Draq-5 (blue). Negative controls (NC) in PBS or IgG are shown in d, h, l, and p. The images were captured using confocal microscopy and a 20x (a plan-Apochromatic) objective lens. LE = luminal epithelium; L= lumen; ST= stroma. Bar = 100 µm (same scale for all micrographs, and 200 µm for insets).

**Figure 4:**
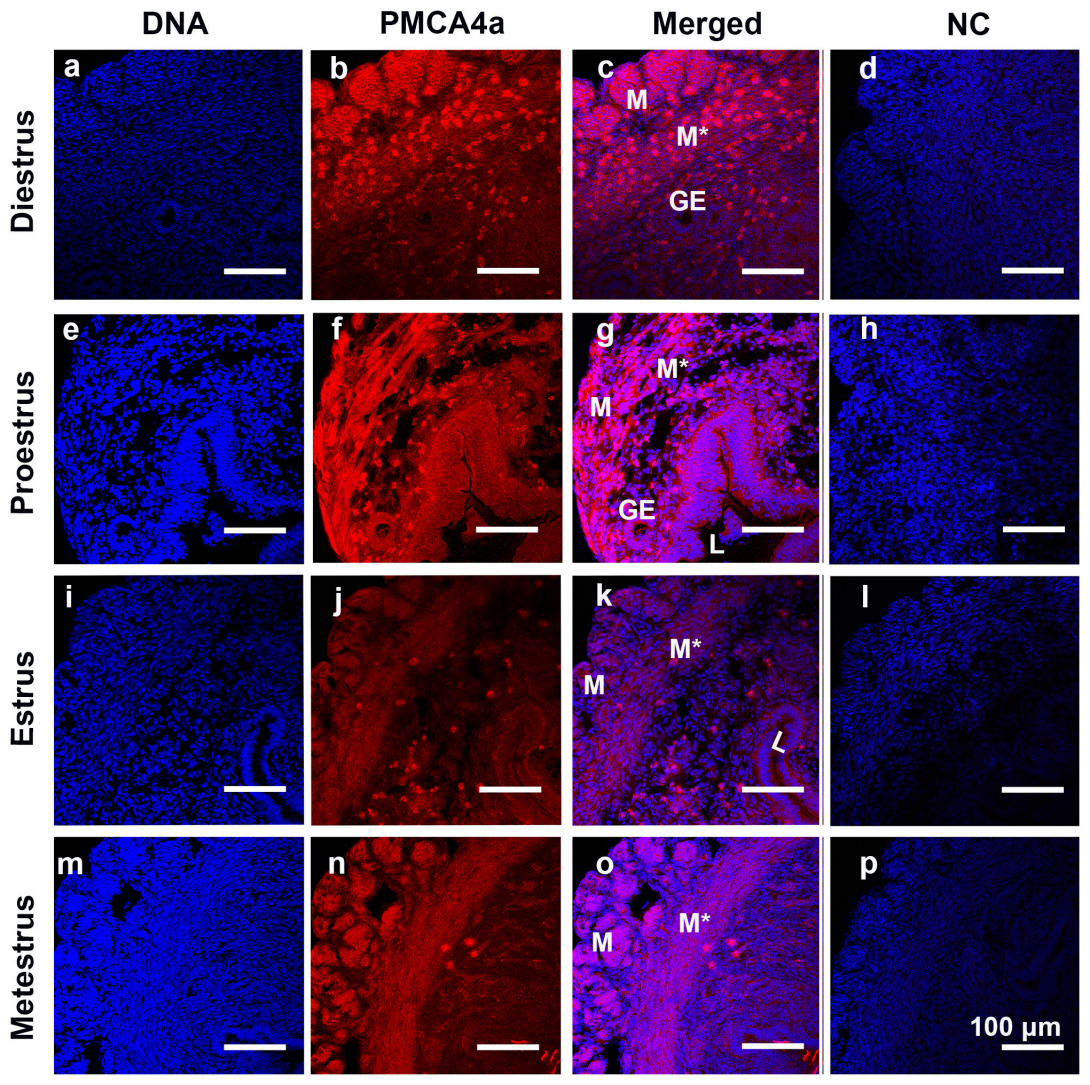
Indirect Immunofluorescence of PMCA4a in the murine myometrium of the uterus during the estrous cycle In addition to the endometrium, the muscles and mesothelium of the myometrium (M*, M, respectively) were positively stained for PMCA4a. Elevated levels of PMCA4a immunoreactivity were detected at the boundaries of the epithelial cells lining the pro-estrus uterine glands (g). The nuclei were visualized by staining with Draq-5 (blue). Negative controls (NC) of diestrus, pro-estrus, estrus, and metestrus phases are respectively shown (d, h, l, and p). The images were captured using confocal microscopy and a 20x (a plan-Apochromatic) objective lens. GE = glandular epithelium; L = lumen; ST= stroma. Bar = 100 µm (same scale for all micrographs).

The uterine expression pattern of PMCA4a in naturally cycling females was determined after vaginal smears were examined to identify the stages of the estrus cycle. Uterine tissues collected from virgins at the four stages of the estrus cycle were analyzed. Interestingly, only in the pro-estrus and estrus phases was expression of PMCA4a detectable in both the luminal and glandular epithelium, while it was absent or marginally present in the metestrus and diestrus phases in these regions (Figure 3, insets). In contrast, the myometrium showed strong expression of PMCA4a in all four phases of the estrus cycle (Figure 4). 

**Figure 5:**
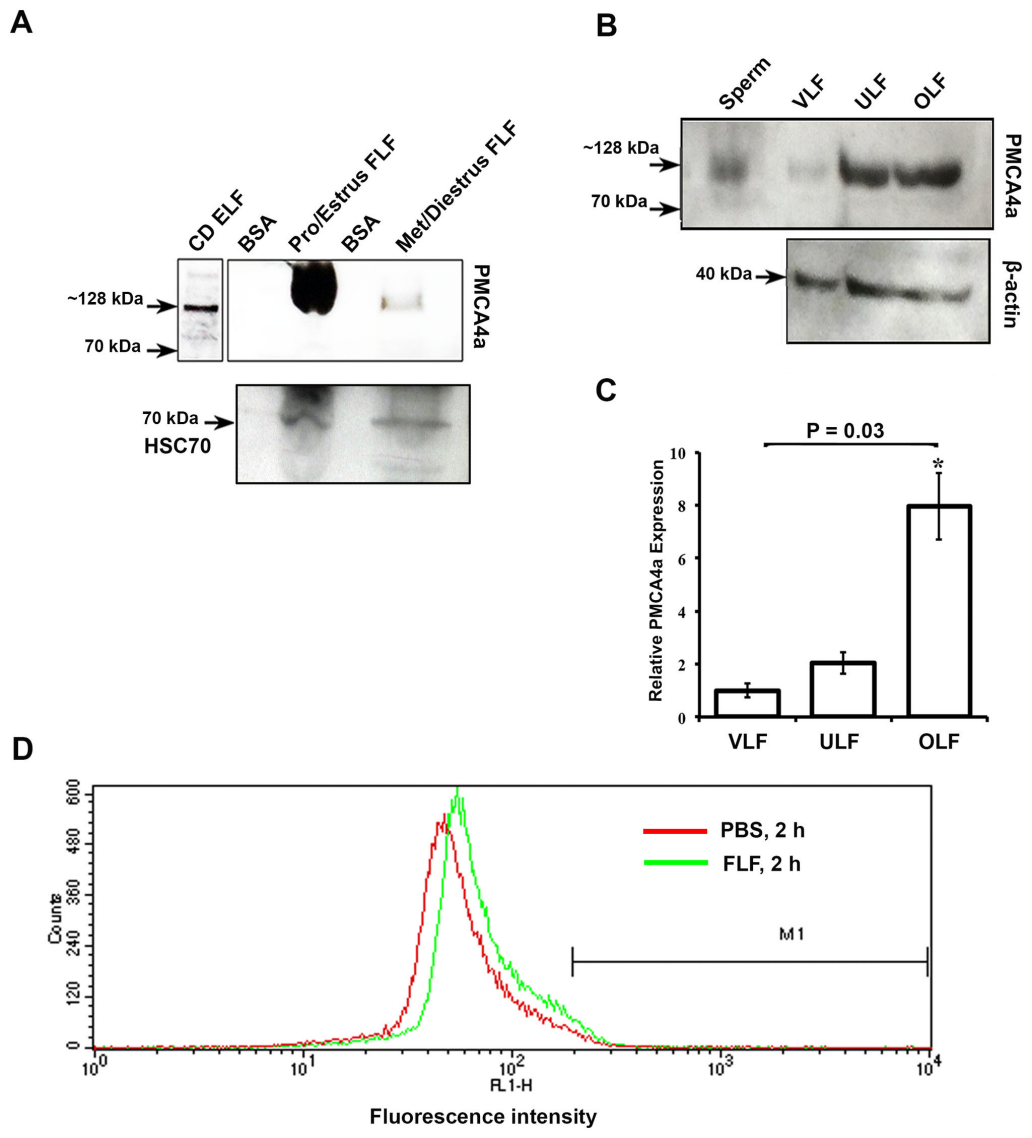
Detection of PMCA4a in reproductive luminal fluids and its acquisition on caudal sperm **A**) Representative Western blot of FLFs collected during pro-estrus and estrus and metestrus and diestrus (40 µg proteins loaded). The ~128 kDa PMCA4a is seen in pro-estrus and estrus and is marginally present at metestrus and diestrus. Caudal epididymal luminal fluid was used as a positive control. The membrane was stripped and re-probed for HSC70 as a loading control. **B**) Western blots of VLF, ULF, and OLF recovered after superovulation demonstrate the presence of the ~128 kDa PMCA4a. Sperm protein was used as a positive control. The membrane was stripped and re-probed for β-actin as a loading control. **C**) Quantitation of Western blot data shown in B; the relative expression was determined using VLF as 1. The data represent the mean (±SEM) of a minimum of three independent experiments, and the intensity was quantified by Image J software. ANOVA and *t*-tests were performed on the mean and *P* values were calculated. *P = 0.03 indicates a significantly increase amount of PMCA4a in OLF compared to that in VLF. **D**) A peak shift of fluorescence intensity to the right, indicates increase amounts of PMCA4a in sperm incubated in FLF compared to PBS for 2 h and treated as described in Materials and Methods.

To confirm the IF data, we performed Western blotting analysis on tissues recovered after superovulation. In all three regions of the female reproductive tract the ~128 kDa PMCA4a band was detected (Figure 2B), corroborating the IF findings. Western analysis also revealed that while females in proestrus/estrus secreted PMCA4a in the luminal fluids combined (FLF) those in metestrus/diestus had only marginal levels of the protein (Figure 5A). As expected, females brought into estrus by superovulation also displayed PMCA4a in the luminal fluids from the vagina (VLF), uterus (ULF), and the oviduct (OLF), as shown in Figure 5B. Densitometric analysis showed that the level of expression of PMCA4a in the LFs varied, being lowest in the VLF and highest in the OLF which is present at the site of fertilization. The 9-fold higher level of PMCA4a in OLF over VLF (Figure 5C) represents a significant increase (*P* = 0.03). 

### Characterization of membrane vesicles from Oviductal Luminal Fluids as Exosomes

**Figure 6:**
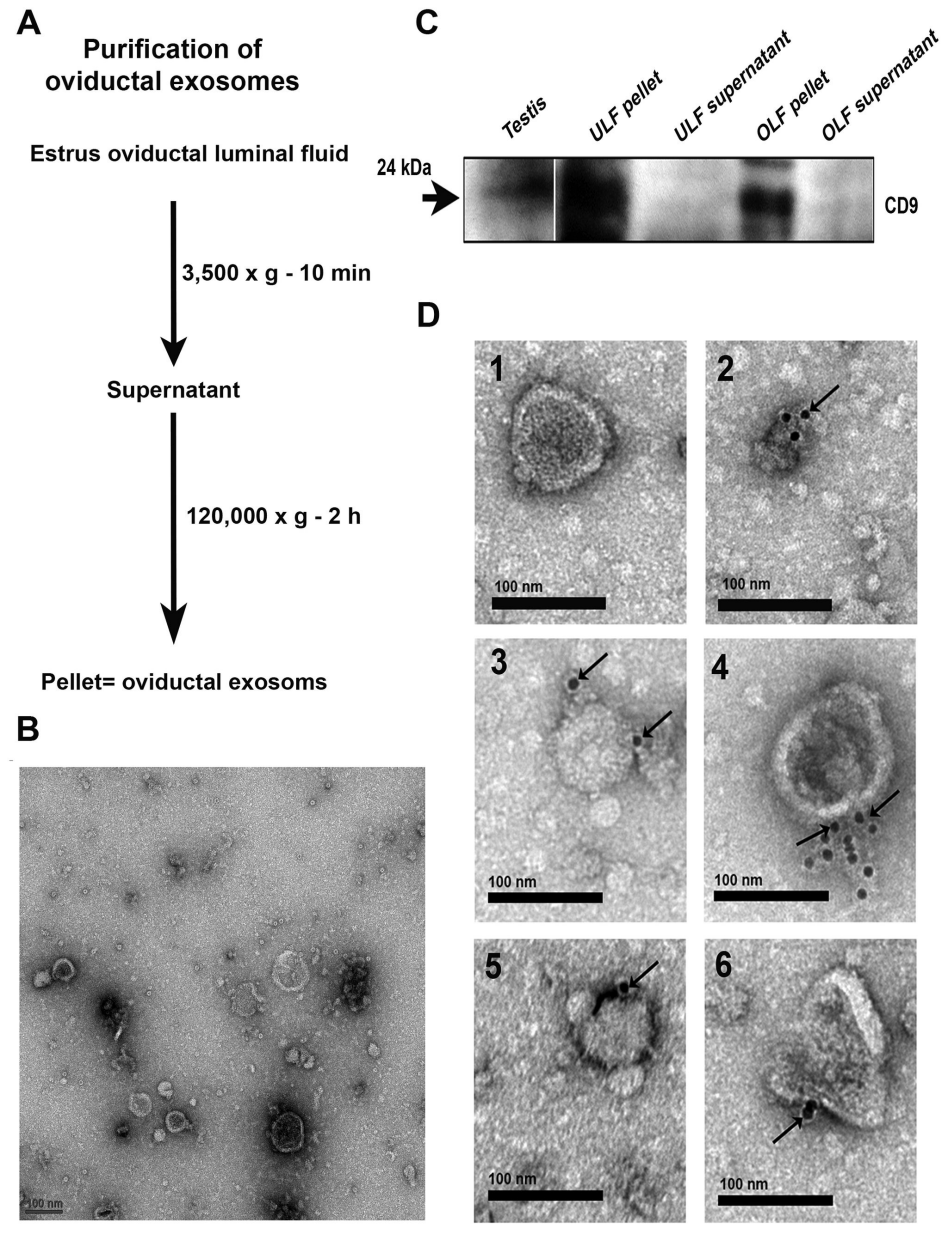
Characterization of membranous vesicles in OLF. **A**) Protocol used to isolate oviductal exosomes by ultracentrifugation of oviductal fluids. **B**) TEM of negative staining for the particulate fraction from OLF reveals the presence of membranous vesicles ranging in size from 25-100 nm in diameter. **C**) Western blots detected CD9 (24 kDa) in protein extracts from membranous vesicles removed from OLF and uterosomes, but not in the supernatants. Testis protein was used as a positive control. Each lane contains 40 µg of protein. Results are representative of three different experiments. **D**) Immunogold labeling (6 nm gold particles) of CD9 is shown in oviductal membranous vesicles termed “oviductosomes”. Gold particles on individual oviductosomes are seen arrowed in 2-6 on the exterior of the membrane. In the absence of primary antibodies and the presence of rat IgG, gold particles were absent (1), indicating the specificity of the antibody. Scale bar =100 nm in panel B, D.

Using antibodies which recognize the extracellular domains of CD9 tetraspanin, one of the most abundant proteins found on the exosomal membrane [20,21], we detected the presence of the 24 kDa CD9 by Western analysis of the OLF pellets as well as of the uterosomes (Figure 6C). The band was however absent in the supernatant. The presence of CD9 in the OLF membrane vesicles was confirmed by TEM analysis and immunogold labeling on the membrane where the gold particles were seen on the exterior (Figure 6D), an orientation that typifies exosomes [19]. Therefore, consistent with the requirements for defining membrane vesicles as exosomes, we show that vesicles in the OLF: 1) are within a size range of 25-100 nm in diameter, as detected by negative staining and TEM (Figure 6B), 2) enriched with CD9 tetraspanin, a biomarker of exosomes [20,21] (Figure 6C), and 3) have membrane proteins in the exosomal orientation. Thus, we defined these vesicles, purified from oviductal fluid, as exosomes and termed them”oviductosomes”

### PMCA4a is Present in the Exosomes of the Luminal Fluids

**Figure 7:**
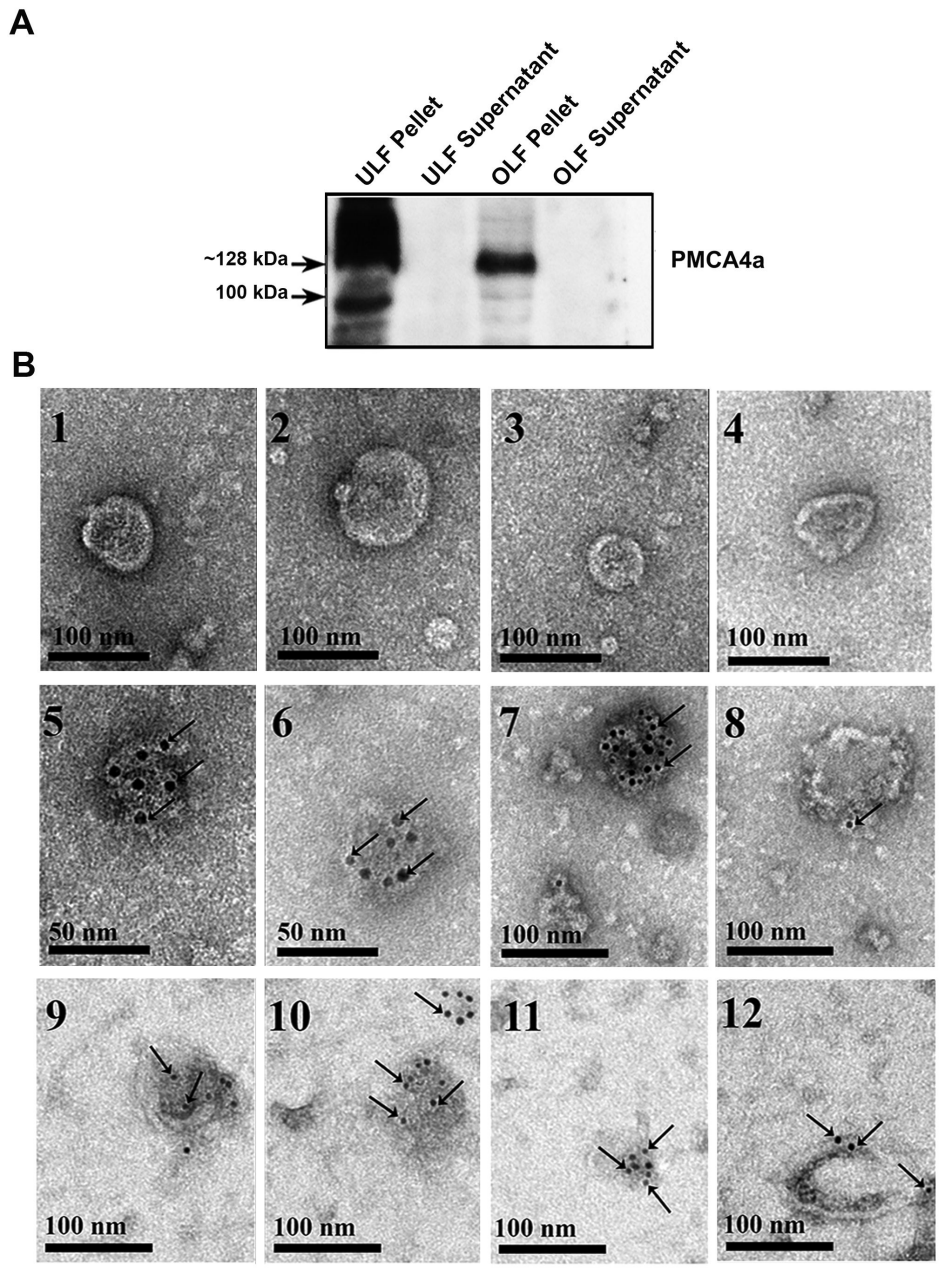
Immunodetection of PMCA4a in oviductosomes and uterosomes. **A**) Nitrocellulose membrane with CD-positive pellets and supernatants (Figure 6C), when stripped and re-probed with PMCA4a antibodies in a Western blot, revealed the presence of the ~128 kDa PMCA4a band in the pellets only. A band of unknown origin at ~100 kDa is also seen in the uterosomes. Each lane contains 40 µg of proteins. Results are representative of three experiments. **B**) Immunogold labeling (6 nm gold particles) of PMCA4a is shown in oviductosomes in 5-8 and in uterosomes in 9-12. Gold particles localized on the cytoplasmic-side of the membrane are seen arrowed. They were rarely seen elsewhere on the grids. In the absence of primary antibodies and the presence of rabbit IgG, gold particles were absent (1-4) indicating the specificity of the antibody. Scale bar= 50-100 nm.

When the nitrocellulose membranes used for the detection of CD9 in the exosomes from ULF and OLF (Figure 6C) were re-probed with anti-PMCA4a antibodies in Western blotting analysis, the ~128 kDa PMCA4a band was detected, but was absent from the supernatant (Figure 7A). For the uterosomes (10), there was a cross-reactive ~100 kDa band that was not seen in the OLF and whose origin is unknown. Our findings indicate that 100% of PMCA4a in the luminal fluids resides in the particulate fraction, or the exosomes. More importantly, our results indicate that PMCA4a is carried on CD9-positive exosomes. 

**Figure 8:**
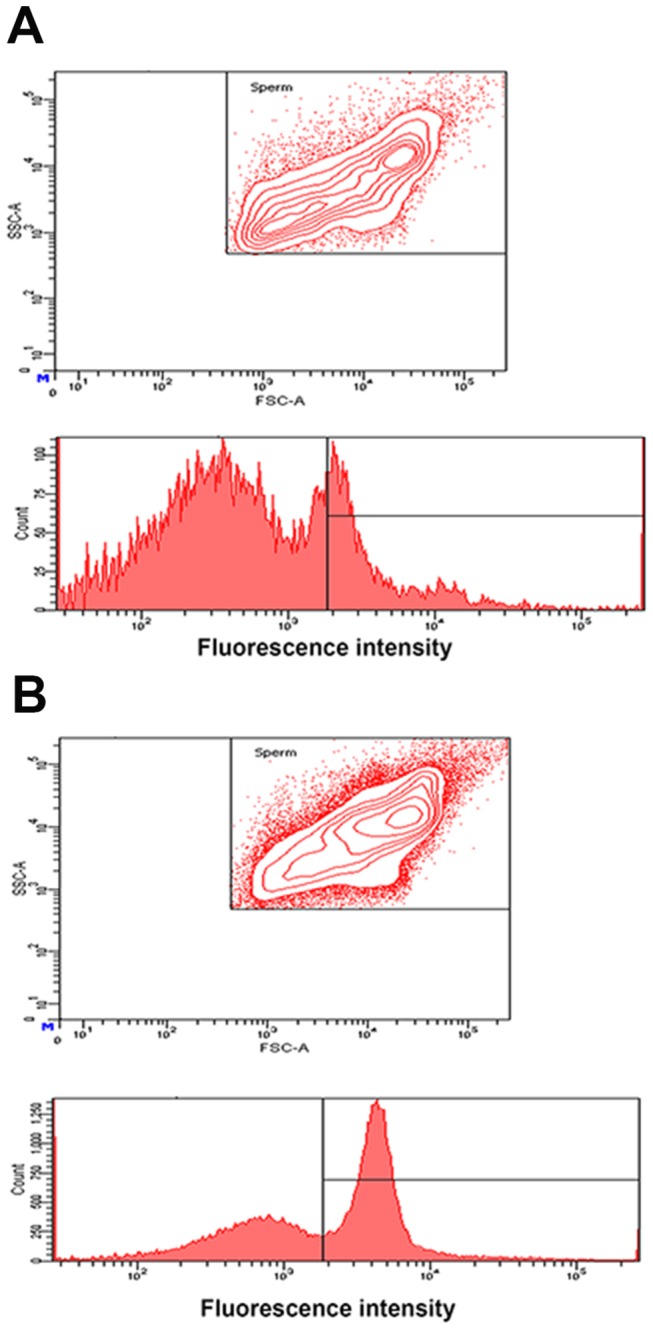
PMCA4a uptake by caudal sperm via incubation in exosomes reconstituted in PBS Exosomes were isolated from FLFs after superovulation. **A**) Flow cytometric analysis of sperm co-incubated in PBS (negative control). The contour plot above reveals two subpopulations of sperm, as represented by the small inner circles at top right and bottom left. The graph shows that the majority of the fluorescence falls below the gated region. **B**) Sperm incubated in FLF exosomes (2 mg/ml protein) show a unimodal distribution (a single inner circle) and a ~3 fold increase of fluorescence intensity (peak shift to the right) compared to **A** in the gated region. The co-incubation period was 3 h for both A and B.

We confirmed the presence of PMCA4a on CD9-positive exosomes by TEM and immunogold labeling. Control samples incubated in the presence of rabbit IgG showed randomly dispersed 6 nm immunogold particles on the grids, but none localized to vesicles (Figures 7B, 1-4). In test samples treated with the primary antibody, immunogold particles detecting PMCA4a were observed on both the oviductosomes (Figures 7B, 5-8) and the uterosomes (Figures 7B, 9-12). It should be noted that gold particles were not randomly distributed and were rarely localized outside of the vesicles on the grids. Importantly, on the vesicles, the particles detecting the localization of PMCA4a (Figure 7B) required gentle permeabilization of the vesicles in order for the PMCA4a antibodies to gain access to, and recognize an epitope on, the cytoplasmic-side of the molecule. This further confirms that the oviductosomes are exosomes, and not endosomes which have the cytoplasmic side of the molecule in the exterior orientation [19].

### *In Vitro* Uptake of PMCA4a on Caudal Sperm from Unfractionated Luminal Fluids and Exosomes

The presence of PMCA4a in the luminal fluid during estrus, the only phase when sperm traverse the female tract, suggested that the pump may be transferred to the sperm surface during their transit and storage in the oviduct. Fresh caudal epididymal sperm were incubated for 2-3 h in PBS supplemented with Zn^2+^ (carrier control), which is known to prevent the acrosome reaction [22], or in FLF suspended in PBS containing Zn^2+^ (added in the form of zinc acetate). Sperm were then fixed and stained with anti-PMCA4a primary and Alexafluor-488 conjugated secondary antibodies as described in the Materials and Methods. Flow cytometry, used to quantify fluorescence intensity as a measure of PMCA4a uptake in 50,000 sperm/group, revealed that after 2 h incubation in FLF there was a right peak shift in fluorescence intensity, compared to the PBS control (Figure 5D). After 3 h co-incubation of sperm and reconstituted exosomes, there was a remarkable ~3-fold increase in uptake compared to the PBS control (Figure 8). In general, the flow cytometric data reveal *in vitro* acquisition of PMCA4a on caudal sperm and suggest that the plasma membrane in these cells is not all fully saturated with this transmembrane protein when sperm leave the male.

## Discussion

### Expression of PMCA4a in the murine female reproductive tract

Of the two major splice variants of PMCA4, 4a and 4b, 4a is faster in extruding Ca^2+^ and returning cytosolic Ca^2+^ concentration to resting levels [13]. Since sperm in the oviduct, particularly the AIJ, have high levels of intracellular Ca^2+^ [23], they need effective efflux mechanisms to prevent Ca^2+^ overload in order to maintain their viability. Our mRNA data (Figure 1) revealed that both *Pmca4a* and *4b* isoforms are expressed in reproductive tissues collected from all three regions of the tract in females undergoing estrus. As estrus is the only time when females are receptive to the male and sperm are likely to be present in the tract, the presence of *Pmca4a* and *4b* mRNAs may be related to sperm capacitation. Therefore their presence in the oviduct, where sperm undergo hyperactivation, with the activation of Ca^2+^ influx by CatSper [24], may be physiologically relevant. The RT-PCR results were corroborated by the high level of PMCA4a in the oviductal apical epithelium and in the OLF. This elevated expression of the more efficient PMCA4a may be necessary to maintain Ca^2+^ homeostasis when the demand for efflux is great. 

When PMCA4a protein was examined by IF in naturally cycling females, it was found to be expressed in the uterine myometrium in all four stages of the estrus cycle (Figure 4). This suggests that the pump plays a key role in the maintenance of uterine function by helping to regulate membrane potential and intracellular Ca^2+^. However, during the estrus and pro-estrus phases, unlike metestrus and diestrus, in addition to its presence in the myometrium there was up-regulation of PMCA4a in the uterine endometrium, as seen in the luminal and glandular epithelia. This suggests that the transcription of the *Pmca4* gene and/or the alternative splicing of its primary RNA transcripts are/is under the control of female sex hormones. Indeed, a Blast search of the promoter region of the *Pmca4* gene for the presence of estrogen response elements (EREs) revealed at -313-335 a potential sequence, 5’-GGGCTgacTGACC-3’. Although this sequence contains two mismatches compared to the consensus ERE [25], it fulfills the requirement for ER-ERE binding for estrogen responsive genes [25]. Further studies will be needed to determine the mechanism of estrogen–mediated up-regulation of PMCA4a expression in the female reproductive tract.

 To study PMCA4a during estrus, virgins were superovulated and sections of all three regions of the female tract analyzed. IF revealed that in addition to the uterus, the vagina and the oviduct showed high expression of PMCA4a. Importantly, all three ductal regions had the strongest PMCA4a staining on the luminal side of the apical membrane of the epithelium. This finding parallels that seen in the epididymis [7] and is consistent with the secretion of PMCA4a from these tissues into the lumen, similar to the secretion of other PMCAs in other tissues [26-29]. 

Western analysis corroborated the IF results and revealed the presence of the ~128 kDa PMCA4a band in all female reproductive tissues as well as in the luminal fluids. Interestingly, in naturally cycling females while fluids collected during metestrus/diestrus had only marginal levels of PMCA4a, those collected during proestrus/estrus had elevated levels of the protein, as seen after induction of superovulation. Here, PMCA4a was most abundant in OLF. These findings are similar to that for SPAM1, a GPI-linked sperm protein which was found to be present on uterosomes, vesicular particles in the uterine fluid [30] during estrus. However, unlike GPI-linked proteins that are found in both the particulate and the soluble fraction of the LF [11,12], we found PMCA4a exclusively in the particulate fraction, the oviductosomes and the uterosomes. This localization is consistent with the PMCA4a’s transmembrane structure. 

Oviductosomes were identified for the first time, and characterized as exosomes based on negative staining, size (25-100 nm), membrane orientation (cytoplasmic-side inward), and the CD9 biomarker. These oviductosomes are likely to originate as internal vesicles of multivesicular bodies (MVBs) with a unique orientation (cytoplasmic-side inward) [19,31]. It is also possible that they could result from an apocrine pathway resulting from blebbing of the apical membrane of the epithelial lining, as reported for epididymosomes in the male reproductive tract [32]. Whatever their origin, they are likely to play a role in cell-cell communication.

### Transport of PMCA4a from the Female Reproductive Luminal Fluids to Sperm during Capacitation

Exosomes are thought to be the vehicle of transport of proteins from luminal fluids to the sperm surface [33,34]. Accordingly, we detected the transfer of PMCA4a from unfractionated FLF and from exosomes to caudal sperm *in vitro* with increases of up to ~3-fold. Our finding strongly suggests that PMCA4a is acquired by sperm *in vivo* during their transit in the female. Figure 8 provides some initial insight into the mechanism of uptake, as we detected that exosomes are not only necessary but are sufficient for the transfer of PMCA4a to the sperm. The presence of PMCA4a’s uptake from reconstituted exosomes, in the absence of soluble proteins in the FLF, is supportive of this conclusion. 

Studies on the mechanism(s) by which vesicles transport proteins to the sperm surface are limited. Griffiths et al. [12,18] have shown that vesicles dock on the sperm membrane and have postulated that hydrophobic interactions may underlie the transfer of GPI-linked proteins which are attached to the outer leaflet of the lipid bilayer of the membrane. However, this mechanism is unlikely to be involved in the transfer of transmembrane proteins, such as PMCA4a which has its catalytic domain on the cytosolic side of the membrane [35]. In the study by Griffiths et al. [12], the data revealed that the vesicles fuse with the sperm membrane, although the authors did not comment on this. It is likely that such sperm-vesicle fusion is the mechanism by which PMCA4a is transferred to sperm. Recently, Caballero et al. [20] have shown that CD9-positive microvesicles mediate the transfer of molecules to bovine sperm during epididymal maturation. It is likely that the CD-9 positive oviductosomes and uterosomes might also play this role in mammalian sperm. Further studies will be required to elucidate the precise mechanism involved.

Whatever mechanism is involved, the *in vitro* uptake of PMCA4a on caudal sperm after co-incubation with female LF indicates that the sperm membrane is not saturated with this protein after epididymal maturation. During progressive maturation in the female, sperm acquire their fertilizing ability while transiting the oviduct [36] where they are stored in the sperm reservoir compartment and remain viable until ovulation occurs [2]. During this period they interact with the oviductal epithelial lining and the oviductal secretions [23,37] and their viability is maintained. To date, very little is known of the component(s) of the secretion that are responsible for sperm viability and maturation. The present study has identified PMCA4a, a key element of the Ca^2+^ handling toolkit, as one of these components and suggests that it is highly likely to play a role in the final maturation of sperm in the oviduct.

 Of interest, the distribution of PMCA4a in the FLF is most abundant in OLF. This is consistent with an increased volume of OLF during the estrus phase [1] when sperm are likely to be present and undergoing hyperactivated motility. Since the latter is accompanied by activation of Ca^2+^ influx via the CatSper channel [24,38], PMCA4a, and likely PMCA4b, could play an essential role in maintaining Ca^2+^ homeostasis and sperm viability. Indeed, deletion of the PMCA4a and 4b pumps causes loss of hyperactivated motility and infertility in mice [4,5] and reduced PMCA4 activity in *Jam-A* null mice has a similar effect [6,39]. On the other hand, lethal calcium concentrations were diminished when bovine sperm were exposed to the apical plasma membrane of oviductal epithelial cells [40]. The authors concluded that anchored proteomic factors on the oviductal epithelial cells are responsible for maintaining sperm motility and viability [40]. The present study now allows us to identify PMCA4a as a potential major proteomic factor involved in diminishing toxic Ca^2+^ levels in bovine sperm co-incubated with oviductal epithelial cells. Thus further studies will focus on whether or not PMCA4a that is transferred to sperm has a functional impact. 

Although there is evidence that epididymal PMCA4a is acquired *in vivo* by sperm [7], it is unknown if this is the case for oviductal PMCA4a. It is also unknown how acquisition of epididymal PMCA4a impacts sperm function. Although *Pmca4* null females are not sterile their level of fertility, compared to WT, has never been investigated. In this vein, the fertilizing competence of *in vitro* capacitated sperm in the presence/absence of oviductal or uterine PMCA4 has yet to be studied. *Pmca4* null mice provide the opportunity for investigating the impact of sperm acquisition of PMCA4 from both the male and female reproductive tracts on Ca^2+^-ATPase activity and ultimately fertilizing competence, investigations which are underway in our laboratory.

 In conclusion our study reveals for the first time the expression and secretion of PMCA4a in the female reproductive tissues and luminal fluids during estrus. The secretion occurs via exosomes from which PMCA4a, and likely 4b, can be acquired by sperm. The acquisition of this important Ca^2+^ handling tool, primarily from the oviductal fluid via oviductosomes which are identified for the first time, is likely to be central to the maintenance of Ca^2+^ homeostasis and sperm viability during their storage in the oviduct and during capacitation and the acrosome reaction, both of which are important steps in fertilization.
